# Addressing COVID-19 in humanitarian settings: a call to action

**DOI:** 10.1186/s13031-020-00307-8

**Published:** 2020-09-10

**Authors:** Jude Alawa, Nawara Alawa, Adam Coutts, Richard Sullivan, Kaveh Khoshnood, Fouad M. Fouad

**Affiliations:** 1 Stanford University School of Medicine, Stanford, California USA; 2 Department of Pediatrics, Baylor College of Medicine, Houston, Texas USA; 3 Department of Sociology, University of Cambridge, Cambridge, UK; 4 Institute for Cancer Policy and Conflict & Health Research Group, King’s College London, London, UK; 5 Yale School of Public Health, New Haven, Connecticut USA; 6 Department of Epidemiology and Population Health, Faculty of Health Sciences, American University of Beirut, Beirut, Lebanon

**Keywords:** COVID-19, Refugees, Internally displaced persons, Humanitarian settings: WASH, Infectious disease

## Abstract

Refugees and internally displaced persons in humanitarian settings are particularly susceptible to the spread of infectious illnesses such as COVID-19 due to overcrowding and inadequate access to clean water, sanitation, and hygiene facilities. Countries facing conflict or humanitarian emergencies often have damaged or fragmented health systems and little to no capacity to test, isolate, and treat COVID-19 cases. Without a plan to address COVID-19 in humanitarian settings, host governments, aid agencies, and international organizations risk prolonging the spread of the virus across borders, threatening global health security, and devastating vulnerable populations. Stakeholders must coordinate a multifaceted response to address COVID-19 in humanitarian settings that incorporates appropriate communication of risks, sets forth resource-stratified guidelines for the use of limited testing, provides resources to treat affected patients, and engages displaced populations.

## Background

The COVID-19 outbreak has indiscriminately spread across the world, including in countries facing ongoing conflict, protracted humanitarian crises, and large numbers of forcibly displaced individuals. Countries experiencing humanitarian emergencies are particularly vulnerable to the spread of infectious diseases and have limited capacity to confront the challenges associated with containing the spread of COVID-19 and treating existing cases. Despite this reality, aid agencies, international actors, and donor governments have been slow to coordinate a multifaceted response, and few policies have been put forth to develop sufficient test-treat-isolate capacity and to equip medical facilities and health personnel with the resources necessary to care for patients with COVID-19. Although the Global Humanitarian Response Plan for COVID-19 sets forth an important strategic framework to address the virus, countries in humanitarian settings still face immense gaps in their capacity to implement proposed solutions [1]. Without further coordinated global assistance to support context-specific solutions in countries affected by humanitarian crises, there is considerable risk that COVID-19 will devastate vulnerable populations of displaced individuals, continue to spread across borders, and threaten global health security. This call-to-action highlights the challenges of addressing COVID-19 in humanitarian settings and presents key recommendations for national governments, international agencies, and humanitarian responders to control the spread of COVID-19 and treat severely infected patients given limited resources.

## Main text

### Heightened risks of the impact of COVID-19 in crisis settings

Despite global calls to support low-to-middle income countries in addressing COVID-19, little attention has been given to refugees and internally displaced persons (IDPs) in humanitarian contexts, particularly those living in camps or informal settings. Refugees and IDPs are often forced into settlements with high population densities, and these overcrowded areas tend to have limited public health services, poor sanitation and waste disposal facilities, and inadequate access to clean water – making them particularly susceptible to a COVID-19 outbreak. According to the United Nations High Commissioner for Refugees, approximately 84% of the 70 million individuals who have been forced to flee their homes are hosted by low-to-middle income countries with weak health systems and water, sanitation, and hygiene (WASH) tools [2]. Of the countries with reported cases of COVID-19, over thirty play host to registered refugee populations of over 20,000 people, in addition to a considerable number of unregistered refugees [3]. Approximately 80% of refugees seek work or reside in urban areas, and refugees or humanitarian responders who travel from overcrowded settlements to cities to seek healthcare or work could worsen the spread of the virus [4]. The United Nations has already ordered the evacuation of Moria refugee camp in Lesbos, Greece, which was designed to host 3000 people but now hosts over 20,000 and has been reported to have widespread malnutrition and poor hygiene [5]. In more established camps such as that of Cox’s Bazar in Bangladesh, which hosts half a million Rohingya refugees, it is likely that the spread of COVID-19 will still pose challenges as the management of cases requires referrals to health facilities that are already overstretched.

The scientific community has continued to make advances in understanding the most effective ways to manage severe cases of COVD-19, most recently with the publication of a clinical trial on the use of dexamethasone [6]. It is known that COVID-19 is transmitted via aerosolized particles, larger droplets, and sustained contact with contaminated surfaces and that severe cases often result in an overwhelming inflammatory response affecting the lungs. Data from affected countries suggest that age (> 65), one or more serious comorbidities, particularly cardiovascular disease or uncontrolled diabetes, and smoking are correlated with increased rates of mortality [7, 8]. Data from the Centers for Disease Control and Prevention also show that approximately 55% of patients aged 20–64 are at risk for moderate to severe infection and require respiratory support in the form of oxygen via nasal cannula, positive pressure ventilation, or in the worst cases, mechanical ventilation [9]. Despite these daunting statistics, the majority of COVID-19 cases are asymptomatic or present with mild symptoms, such as a cough and congestion. This trend is expected to hold true among refugees and IDPs as well. However, it is not yet clear if there are particular health and social characteristics specific to these vulnerable groups which will result in higher rates of transmission and mortality, especially among younger age groups. The actual burden of COVID-19 in areas hosting refugees and IDPs is still unknown given a serious lack of testing capabilities. Due to overcrowding, poor WASH tools, and limited availability of advanced health services, it is expected that rates of transmission and mortality will be higher in humanitarian settings. In Somalia, documentary sources claim that nearly 70% of daily tests are returning positive as the country surpasses 3000 reported cases [10].

For severely infected patients, hospitalization, respiratory support, and even cardiac resuscitation may be necessary, in addition to continued management of chronic comorbid conditions such as cancer and cardiovascular disease that are increasingly prevalent in humanitarian settings [11, 12]. These patients will likely struggle immensely to receive care in such contexts given that the majority of conflict-affected nations with large populations of IDPs and refugees do not have the health infrastructure nor the medical personnel to provide these services. In humanitarian contexts, hospitals have been destroyed; medical personnel have been killed or displaced; and, the provision of medical supplies, including personal protective equipment (PPE) required to prevent COVID-19 transmission, is inadequate [13]. In Syria, for example, only 64% of hospitals and approximately 52% of primary care centers are still functional [14]. It must also be noted that most available research and interventions using anti-viral medications, such as Remdesivir and steroids for cytokine storm, have been developed against a backbone of high-income and high-dependency intensive care, which are rarely available in humanitarian settings.

### Upholding international frameworks and protecting global health security

Many of the nations that host the majority of the world’s refugees are signatories to the 1951 UN Refugee Convention and its 1967 Protocol or similar regional frameworks that state that refugees should receive the same health services as nationals [15]. Given these international commitments and the necessity of treating all affected populations to contain the spread of COVID-19, refugees and IDPs must be included in national response plans. Furthermore, the COVID-19 pandemic can affect anyone, regardless of race, gender, age, or political affiliation. Like all infectious pathogens, COVID-19 has no regard for national borders. Given the transient nature of populations in crisis settings, an inadequate response to COVID-19 in such settings is likely to prolong the spread of the virus and threaten global health security. As such, it is the collective responsibility of global leaders and international organizations to coordinate a robust response to prevent the spread of COVID-19 and treat affected patients in humanitarian settings.

### COVID-19 response priorities in humanitarian settings

There is much that should be done to address COVID-19 in humanitarian settings. First, governments, aid agencies, and international actors must put forth clear prevention and treatment options that are consistent with available resources. To reduce transmission of this highly contagious pathogen, there needs to be effective communication of risks to both affected populations and front-line responders, explaining how the virus is transmitted, the importance of handwashing, sanitation, and hygiene in reducing transmission, and the necessary steps to take if there is concern for infection. Only then can stakeholders work together to further prevent transmission by implementing physical distancing measures, restructuring settlements, and improving the availability of WASH, as dictated by the World Health Organizations response guidelines [16]. Second, given the reality of inadequate laboratory access in crisis settings, resource-stratified guidelines for the use of limited testing must be put forth to identify cases and prevent further transmission. The mistakes of severely affected countries and the successes of countries who have been able to avoid and reduce transmission have taught us that early and broad testing is imperative to controlling the spread of this virus. Until widespread testing is available, governments and humanitarian agencies must provide explicit guidance for the use of limited tests, perhaps prioritizing at-risk populations and healthcare workers. Third, because it is expected that transmission will not be well-controlled in some settings, there must be a context-specific preparedness plan for case management. In addition to ensuring the availability of resources that could be potentially life-saving, such as PPE for healthcare workers, oxygen tanks, nasal cannulas, and ventilators when possible, this is a setting where mobilizing additional healthcare professionals, creating mobile screening sites, and establishing temporary healthcare facilities would be effective in treating a potential surge of acutely ill patients. It may be more practical to have safe wards supporting non-invasive respiratory needs as opposed to sophisticated intensive care technologies that are not likely to be the best use of limited resources. In the worst case, providers in these settings should be ready to employ mass-casualty tagging techniques if spread is rampant and mortality rates increase [17]. If resources are limited and patients are expectant, frank conversations must be had to determine the patient’s goals of care along the spectrum of illness. In spite of the limited availability of treatment resources for COVID-19, however, frontline responders should not divert resources away from already necessary life-saving health services, such as emergency obstetrics and vaccination. Lastly, given the immense demand for health practitioners, the COVID-19 pandemic provides an opportunity to review national healthcare policies and integrate refugees into national health systems, allowing displaced healthcare workers to practice and fill human resource gaps.

## Conclusions

### A call to action

It is well known by now that COVID-19 is highly contagious and will spread quickly in humanitarian settings, particularly in confined and crowded camp settings. In fact, this may be occurring already, but due to limited testing capacity for COVID-19 infection in such settings, there is no way to assess the current burden. Without a strategy to identify cases of COVID-19, to mitigate the spread of infection, and to treat affected patients, the rapid transmission and ultimate death of thousands of people in humanitarian settings becomes an inevitable and unspoken truth of this pandemic [18]. If this was not enough motivation, the spread of infection, particularly in urban areas, is linked to cases among the most vulnerable. While global health agendas and international policies often only effectively protect some, it is clear now, more than ever, that the outcomes of some are inextricably linked to the outcomes of all. Our connectedness is undeniable, and inaction in addressing COVID-19 in humanitarian settings may threaten global health security moving forward. In order to achieve the response priorities highlighted, national governments, international agencies, and humanitarian organizations must promptly collaborate to develop context-specific strategies to promote risk communication, prioritize the use of existing testing kits and increase their overall availability, provide resources to treat affected patients, and engage displaced populations. While addressing COVID-19 in humanitarian settings may appear to be an acute challenge, stakeholders, especially national governments and international donors, can consider commitments to combatting COVID-19 as long-term investments in the success of a given country’s health system and the livelihood of its inhabitants.

## Data Availability

Not applicable.
